# Negative Regulation of Hepatic Inflammation by the Soluble Resistance-Related Calcium-Binding Protein *via* Signal Transducer and Activator of Transcription 3

**DOI:** 10.3389/fimmu.2017.00709

**Published:** 2017-06-29

**Authors:** Xiaying Li, Yanan Liu, Yongqiang Wang, Jue Liu, Xiaoqi Li, Hong Cao, Xiang Gao, Shijun J. Zheng

**Affiliations:** ^1^State Key Laboratory of Agrobiotechnology, Beijing, China; ^2^Key Laboratory of Animal Epidemiology and Zoonosis, Ministry of Agriculture, Beijing, China; ^3^College of Veterinary Medicine, China Agricultural University, Beijing, China; ^4^Institute of Veterinary and Animal Sciences, Beijing Academy of Agriculture and Forestry, Beijing, China; ^5^Model Animal Research Center, Nanjing University, Nanjing, China

**Keywords:** sorcin, signal transducer and activator of transcription 3, negative regulator, NF-κB, immune response, inflammation, sorcin-deficient mouse

## Abstract

Host immune response is tightly controlled by negative regulators to avoid excessive immune reactions for homeostasis. Some pathogens may take advantage of host negative regulating system to evade host defense. Our previous report showed that foot-and-mouth disease virus (FMDV) VP1 inhibited TNF-α- and SeV-induced type I interferon response *via* interaction with cellular protein soluble resistance-related calcium-binding protein (sorcin). Conversely, TNF-α- or SeV-induced type I interferon response increased when sorcin knocked down, leading to inhibition of vesicular stomatitis virus replication. However, the exact role of sorcin in regulation of the immune response is still not clear. Here, we show that mice deficient of sorcin (sorcin^−/−^) display enhanced ConA-induced hepatitis. Importantly, splenocytes from sorcin^−/−^ mice produced more IL-2, IL-4, IL-17, and IFN-γ than that of littermate controls (sorcin^+^^/^^+^) in response to anti-CD3/28 stimulation. Furthermore, our data indicate that sorcin interacts with signal transducer and activator of transcription 3 (STAT3) and enhances its phosphorylation and that STAT3 acts as an immediate downstream molecule of sorcin in the negative regulation of NF-κB signaling. Thus, sorcin, in association with STAT3, negatively regulates hepatic inflammation.

## Introduction

Host immune response is tightly controlled by negative regulators to avoid excessive immune reactions for homeostasis. Some pathogens may take advantage of host negative regulating system to evade host defense and establish persistent infection. Foot-and-mouth disease virus (FMDV) is such a kind of pathogens that regardless vaccinated or not, some animals infected with FMDV experience a long-term asymptomatic infection (1, 2). The mechanism of how this virus establishes persistent infection in host is not very clear. Our recent publication shows that VP1 inhibits TNF-α-induced activation of NF-κB and type I interferon promoters *via* interaction with cellular protein soluble resistance-related calcium-binding protein (sorcin) (3). This observation suggests that sorcin might act as an important component of host negative regulating system that dampens cell response to viral infections.

Lines of evidence showed that negative regulating proteins played an essential role in homeostasis, such as suppressors of SOCS proteins as counterparts for STAT (4, 5), Mdm2 as negative regulator for p53 (6–8), and CTLA4 as a brake for TCR-mediated T cell activation (9–11). Loss of these negative regulators leads to reinless cell responses to stimuli, resulting in varied disorders in animals. Transcriptional regulators NF-κB are involved in multiple physiological processes. Recent evidence indicates that signal transducer and activator of transcription 3 (STAT3) acts as an inhibiting protein for NF-κB (12). Thus, STAT3 may serve as a brake to slow down NF-κB-mediated cell response, which may avoid cell response going out of control.

Sorcin was originally identified in drug-resistant cells (13, 14). Recent evidence show that sorcin interacts with mitochondrial chaperone Trap1 and both are involved in multi-drug resistance, and the intramitochondrial sorcin plays a role in TRAP1 cytoprotection (15). Since Trap1 is involved in antioxidant and anti-apoptosis in multi-drug-resistant cells, sorcin may serve as a cell protective molecule (15). Our previous report showed that sorcin inhibited TNF-α- or SeV-induced activation of type I interferon and NF-κB promoters (3), revealing a novel role of sorcin as an important cellular regulator for host response.

To investigate the role of sorcin in inflammatory response and the underlying molecular mechanisms, we generated mice deficient of sorcin (sorcin^−/−^) and induced hepatitis in sorcin^−/−^ mice with ConA. We found that ConA-induced hepatitis was markedly enhanced in sorcin^−/−^ mice. We also observed that splenocytes from sorcin^−/−^ mice produced much greater IL-2, IL-4, IL-17, and IFN-γ than that of WT controls. Furthermore, our data show that sorcin interacted with STAT3 and enhanced STAT3 phosphorylation upon IL-6 treatment, indicating that sorcin, *via* interaction with STAT3, is involved in negative regulation of hepatic inflammation.

## Materials and Methods

### Mice

Sorcin^−/−^ mice were generated as followed and backcrossed with C57BL/6 or BALB/c mice for over ten generations before use. Age- and sex-matched sorcin^+/+^ littermates were used as controls. Mice were housed in China Agricultural University Animal Care Facilities under SPF conditions.

### Cells

HEK293T and HepG2 cells were obtained from ATCC. All the cells were cultured in DMEM/HEPES/pyruvate supplemented with 10% FBS in a 5% CO_2_ incubator at 37°C.

### Reagents

All restriction enzymes were purchased from NEB (USA). Endo-toxin Free Plasmid Preparation Kit, EASYspin plus RNA extraction Kit were purchased from Aidlab (China). PrimeScript RT reagent Kit was purchased from TaKaRa (Japan). Anti-actin (SC-130656), anti-sorcin (SC-100859), and anti-GFP (SC-9996) antibodies and protein A/G plus-agarose (SC-2003) were purchased from Santa Cruz Biotechnology (USA). Anti-FLAG M2 (F1804) antibody was purchased from Sigma-Aldrich (USA). Anti-pSTAT3 (Tyr705) (ab76315) was purchased from Abcam (USA). Anti-STAT3 (7907) and anti-pSTAT3 (Ser727) (9134), were purchased from cell signaling technology (USA). FITC-conjugated goat anti-mouse IgG, TRITC-conjugated goat anti-rabbit IgG, and HRP-conjugated goat anti-mouse and anti-rabbit IgG antibodies were purchased from DingGuoShengWu (China). DMEM, OPTI-MEM I, RNAiMAX, and LTX were purchased from Invitrogen (USA). Transfection reagent Vigofect was purchased from Vigorous (China). Transfection reagent jetPRIME™ was purchased from Polyplus-transfection Biotechnology Company (France). DAPI was purchased from Beytime Company (China). Protease inhibitor cocktail C was purchased from Yataihengxin Company (China). Dual-Luciferase Reporter Assay System was purchased from Promega (USA). Recombinant Human TNF-α was purchased from PeproTech company (USA). Enhanced chemiluminescence (ECL) kit was purchased from Kangwei Biological Company (China). CBA kits were purchased from BD Biosciences Company (USA). Aspartate aminotransferase (AST) and ALT activity assay kits were purchased from Nanjing Jiancheng Company (China).

### Constructs

NF-κB promoter luciferase reporter plasmids were kindly provided by Drs. Hongbin Shu and Youhai Chen. pDsRed-monomer-N1 and pEGFP-N1 vectors were purchased from Clontech (USA). pRK5-flag vector was kindly provided by Dr. Shimin Hu. Human *sorcin* was originally cloned from HEK293T cells by RT-PCR using the specific primers (Sense: 5′-ATGGCGTACCCGGGGCATC-3′; Anti-sense: 5′-TTAAACACTCATGACACATTGAATGAAATC-3′) that were designed according to the gene sequence of *sorcin* in GenBank (Access ID: NM_003130.3). The pRK5-flag-*sorcin*, pEGFP-*sorcin*, and pDsRed-*sorcin* expression plasmids were constructed by standard molecular biology techniques. Human *STAT3* was originally cloned from HEK293T cells by RT-PCR using the specific primers (Sense: 5′-ATGGCCCAATGGAATC-3′; Anti-sense: 5′-TCACATGGGGGAGGTAGCGC-3′) that were designed according to the gene sequence of *STAT3* in GenBank (Access ID: NM_139276.2). The pRK5-flag-*STAT3* and pEGFP-*STAT3* expression plasmids were constructed by standard molecular biology techniques. All the primers were synthesized by Sangon Company (Shanghai, China).

### Generation of Sorcin-Deficient Mice

Sorcin-deficient mice were generated by the conventional gene knockout strategy. There are two different transcription isoforms composed of eight exons for mouse *sorcin* gene (Chromosome 5: 8,046,078–8,069,379). The isoforms have different exon 1, but share exons 2–7. Exons 3–6 were deleted by replacing Exon 3–6 (~4.5 kb) with a loxp-pgkneo-loxp cassette. TL1 embryonic stem cells from 129S6/SvEvTac mice were electroporated with the targeting vector and subjected to positive and negative selection using G418 and ganciclovir, respectively. Two ES cell clones were identified by Southern Blot, which had a copy of the *sorcin* gene replaced by the neomycin resistance gene cassette. The mutant ES cells were injected into 4-day-old C57BL/6J mouse blastocysts. The resultant chimeric male offsprings were crossed with 129S6/SvEvTac females for germline transmission. Unless indicated otherwise, all mice used in this study were of the C57BL/6 background. Mice were housed in China Agricultural University Animal Care Facilities under SPF conditions.

### Induction and Evaluation of ConA-Induced Hepatitis

Seven- to eight-week-old male sorcin-deficient mice and their littermates as controls were injected *via* the tail vein with a single dose (20 mg/kg of body weight) of ConA (Sigma-Aldrich, St. Louis, MO, USA). Blood was obtained through orbital plexus bleeding, and alanine aminotransferase (ALT) and AST levels were determined using AST and ALT activity assay kit.

### Histochemistry Analysis

The livers were removed, and part of the tissues were fixed in 4% phosphate-buffered paraformaldehyde and embedded in paraffin. Tissue sections (5 µm) were prepared and strained with hematoxylin and eosin. Sections were then examined under light microscopy. A total of 10 tissue sections were analyzed for each sample. The degree of hepatic inflammatory infiltrates was scored as follows. Briefly, lobular inflammation was graded into 0–4 based on the numbers of inflammatory foci per field (100×): 0 (grade 0), 1 (grade 1), 2 (grade 2), 3–4 (grade 3), and >4 (grade 4).

### Splenocyte Isolation

Seven- to eight-week-old male sorcin-deficient mice and their WT littermates as controls were sacrificed, and the spleens were surgically removed. Splenocytes were prepared with reference to the Current Protocol of Immunology (16) and cultured in DMEM/HEPES/pyruvate supplemented with 10% FBS in a 5% CO_2_ incubator at 37°C.

### Cytokine Examination

Splenocytes were cultured at a density of 2 × 10^6^ per well in U-bottom 96-well plates containing in the presence of various concentrations of plate-bound 5 µg/mL anti-CD3 mAb and 2 µg/mL soluble anti-CD28 mAb or 2.5 µg/mL ConA. After 48 h, the culture supernatants were collected and cytokine concentrations were determined by commercial CBA kits per the manufacturers’ instructions.

### MTT Assay

Splenocytes were cultured at a density of 5 × 10^5^ per well in U-bottom 96-well plates containing 5 µg/mL plate-bound anti-CD3 mAb and 2 µg/mL soluble anti-CD28 mAb or 2.5 g/mL ConA. Forty-eight hours after culture, culture medium was carefully removed and changed for a fresh one. MTT solution (5 mg/mL PBS, Sigma-Aldrich, St. Louis, MO, USA) was then added at 96 h, and the plate was placed at 37°C for 4 h, followed by treatment with DMSO. The metabolically active cells reduced MTT to blue formazan crystals. MTT-formazan crystals were dissolved for 15 min in DMSO and absorbance was measured at 490 nm plate reader (TECAN, Switzerland).

### Yeast Two-Hybrid Screen and Colony-Life Filter Assay

Yeast two-hybrid screen was performed according to the manufacturer’s protocol (Matchmaker Two-Hybrid System 3). The pGBKT7-*sorcin* plasmid expressing the fusion protein GAL4-BD-sorcin was used as bait and transformed into *Saccharomyces cerevisiae* AH109 which is Trp^−^ and contains HIS3, ADE2, and LacZ reporter genes for GAL4 transcriptional activity. Human cDNA expression library fusion to the GAL4 activation domain in the pGADT7 plasmid was transformed into the *Saccharomyces cerevisiae* strain Y187 which is Leu^−^ and contains the lacZ reporter genes. In β-Gal colony-life filter assay, the bait plasmid was shown to have no transactivation activity. The cDNA Library clones expressing the interacting prey proteins were screened by yeast mating. Positive clones were selected on SD/-Ade/-His/-Leu/-Trp medium and tested for β-gal activity (LacZ^+^) by colony-lift filter assay. Yeasts transfected with pGBKT7-*p53* and pGADT7-*T* were used as positive control (turning blue within 20–30 min), and those transfected with pGBKT7-*Lam* and pGADT7-*T* as negative controls (no turning blue).

The resulting clones were sequenced with the GAL4-AD sequencing sense primer 5′-AGATGGTGCACGATGCACAG-3′ and the results were BLASTed against the NCBI database.

### Cell Culture and Transfection

HEK293T and HepG2 cells were seeded on 6 or 24 plates and cultured in DMEM/HEPES/pyruvate with 10% fetal bovine serum in 5% CO_2_ at 37°C. Twenty-four hours later, cells were transfected with plasmids using transfection reagents Vigofect or LTX per the manufacturer’s instruction.

### Immunoprecipitation and Western Blot

The HEK293T cells (8.0 × 10^5^/well) were seeded on 6-well plates and cultured overnight before transfection with 2.5 µg of pRK5-flag-*STAT3* or pRK5-flag, and pEGFP-*sorcin* or pEGFP-N1. Twenty-four hours after transfection, cell lysates were prepared with 0.2 mL lysis buffer (1% Triton-100, 20 mM Tris–Cl, 5 mM EDTA, 137 mM NaCl, 0.02% NaN_3_, and 1% protease inhibitor cocktail C) at 4°C for 30 min, then centrifuged at 13,000 × *g* for 20 min and the supernatants were saved at −70°C for future use. For immunoprecipitation, cell lysates were incubated with 2 µg of anti-flag antibody at 4°C for 2 h and then mixed with 20 µL of 50% slurry of Protein A/G Sepharose and incubated for another 2 h. Beads were washed three times with lysis buffer and boiled with 2 × SDS loading buffer for 10 min. The samples were fractionated by electrophoresis on a 12% SDS-PAGE gel and transferred onto PVDF membrane. After blocking with 5% skimmed milk, the membrane was incubated with either anti-FLAG or anti-GFP antibodies, and with HRP-conjugated secondary antibodies. Blots were developed using an ECL kit.

For endogenous immunoprecipitation, HEK293T cells were seeded on 6-well plates and cultured overnight before transfection with 5 µg of pRK5-flag-*STAT3* or empty vector. The procedure was performed as above described except that cell lysates were immunoprecipitated using anti-FLAG antibody and Western Blot was performed with anti-sorcin and anti-FLAG antibodies.

### Chemical Crosslinking Analysis

HepG2 cells were washed twice with PBS and incubated with 1 mmol/L disuccinimidyl suberate (DSS) (Pierce) at room temperature for 30 min. The crosslinker reaction was quenched by incubating with 20 mmol/L Tris (pH7.4) for 15 min at room temperature, followed by two washes with ice-cold PBS. The cells were lysed in Non-idet-40 lysis buffer before immunoprecipitation with anti-sorcin or anti-STAT3 antibody and Western Blot was performed as above described.

### Luciferase Reporter Assays

HEK293T cells (1.5 × 10^5^/well) were seeded on 24-well plates and transfected with pRK5-flag-*STAT3* or pRK5-flag-*sorcin* plasmids or empty vector (0.5 µg) together with 0.05 µg of reporter gene plasmids using transfection reagent Vigofect per manufacturer’s instruction. To normalize for transfection efficiency, we added 0.01 µg of pRL-*TK* Renilla luciferase reporter plasmid to each transfection. Eighteen hours after transfection, cells were treated with TNF-α at a final concentration of 20 ng/mL or medium as control. Twelve hours after TNF-α treatment, luciferase assays were performed with a dual-specific luciferase assay kit. For the measurement of SeV-induced activation of NF-κB promoters, cells were transfected with pRK5-flag-*STAT3* or pRK5-flag-*sorcin* or empty vector together with indicated reporter gene plasmids as above described. Eighteen hours after transfection, cells were infected with Sendai virus (SeV) at an MOI of 10 or mock-infected as controls. Twenty-four hours after SeV infection, luciferase assays were performed with a dual-specific luciferase assay kit. Firefly luciferase activities were normalized on the basis of Renilla luciferase activities.

All reporter assays were repeated for at least three times. Data shown were average values ± SD from one representative experiment.

### Knockdown of Sorcin and STAT3 by RNAi

The siRNA was designed by Genechem Company (Shanghai, China) and used to knockdown sorcin or STAT3 in HEK293T cells. The sequences of siRNA for targeting *sorcin* in HEK293T cells included: RNAi#1 (sense: 5′-CUGACAACAAUGGGAUUUATT-3′), RNAi#2 (sense: 5′-GUCAAACUGAGGGCUCUUATT-3′), and a negative control (sense: 5′-UUCUCCGAACGUGUCACGUTT-3′). The sequences of siRNA for targeting *STAT3* in HEK293T cells included: RNAi#1 (sense: 5′-UGACCAACAAUCCCAAGAATT-3′), RNAi#2 (sense: 5′-ACAAUCUACGAAGAAUCAATT-3′), RNAi#3 (sense: 5′-AAAGAAUCACAUGCCACUUTT-3′), and a negative control (sense: 5′-UUCUCCGAACGUGUCACGUTT-3′). HEK293T cells (4 × 10^5^/well) were seeded on 6-well plates and cultured for at least 20 h prior to transfection with siRNA using RNAiMAX according to the manufacturer’s instructions. Double transfection was performed at a 24-h interval. Twenty-four hours after the second transfection, cells were harvested for further analysis by Western Blot.

### Immunofluorescent Confocal Microscopy

HepG2 cells (1.0 × 10^5^/well) were seeded on 24-well plates with coverslips and cultured overnight. Twenty-four hours after transfection, the cells were fixed with 1% paraformaldehyde and washed with PBS three times. The nuclei were stained with DAPI. For endogenous protein staining, the fixed cells were permeabilized with 0.1% triton X-100 for 15 min, blocked with 1% bovine serum albumin, and then probed with anti-sorcin mouse monoclonal antibody or anti-STAT3 rabbit monoclonal antibody at room temperature for 1 h. After three washes, the cells were incubated with FITC-conjugated goat anti-mouse antibody or TRITC-conjugated goat anti-rabbit antibody. The cell samples were observed with a laser confocal scanning microscope (Nikon C1 standard detector; Nikon, Japan). The software of FLUOVIEW was used to analyze the results.

### Examination of STAT3 Phosphorylation

Hep2G cells were transfected with pEGFP-*sorcin* or pEGFP-N1 empty vector or sorcin RNAi constructs, and the cells were stimulated with IL-6 (20 ng/mL) for the indicated periods. The cells were lysed, and the cell lysates were examined by Western Blot using anti-pSTAT3 (Tyr705), anti-pSTAT3 (Ser727), anti-STAT3, and anti-GFP or anti-actin antibodies.

### Statistical Analysis

Statistical analyses were done in GraphPad Prism, version 6.0. The significance of the differences between sorcin^−/−^ and controls in spleen weight, AST and ALT levels, and splenocyte proliferation and cytokine expressions and between treatment and controls in the activation of NF-κB promoters and phosphorylation of STAT3 was determined by the two-way ANOVA and Mann–Whitney accordingly.

### Study Approval

All procedures for animal experiments were approved by the Institutional Animal Care and Use Committee of China Agricultural University (Approval IDs: XXMB-2012-03-01-1, XXMBB-2012-03-15-1, and SKLAB-2016-01-06) and used in accordance with regulations and guidelines of this committee.

## Results

### Enhanced ConA-Induced Hepatitis in Sorcin-Deficient Mice

**Figure 1:**
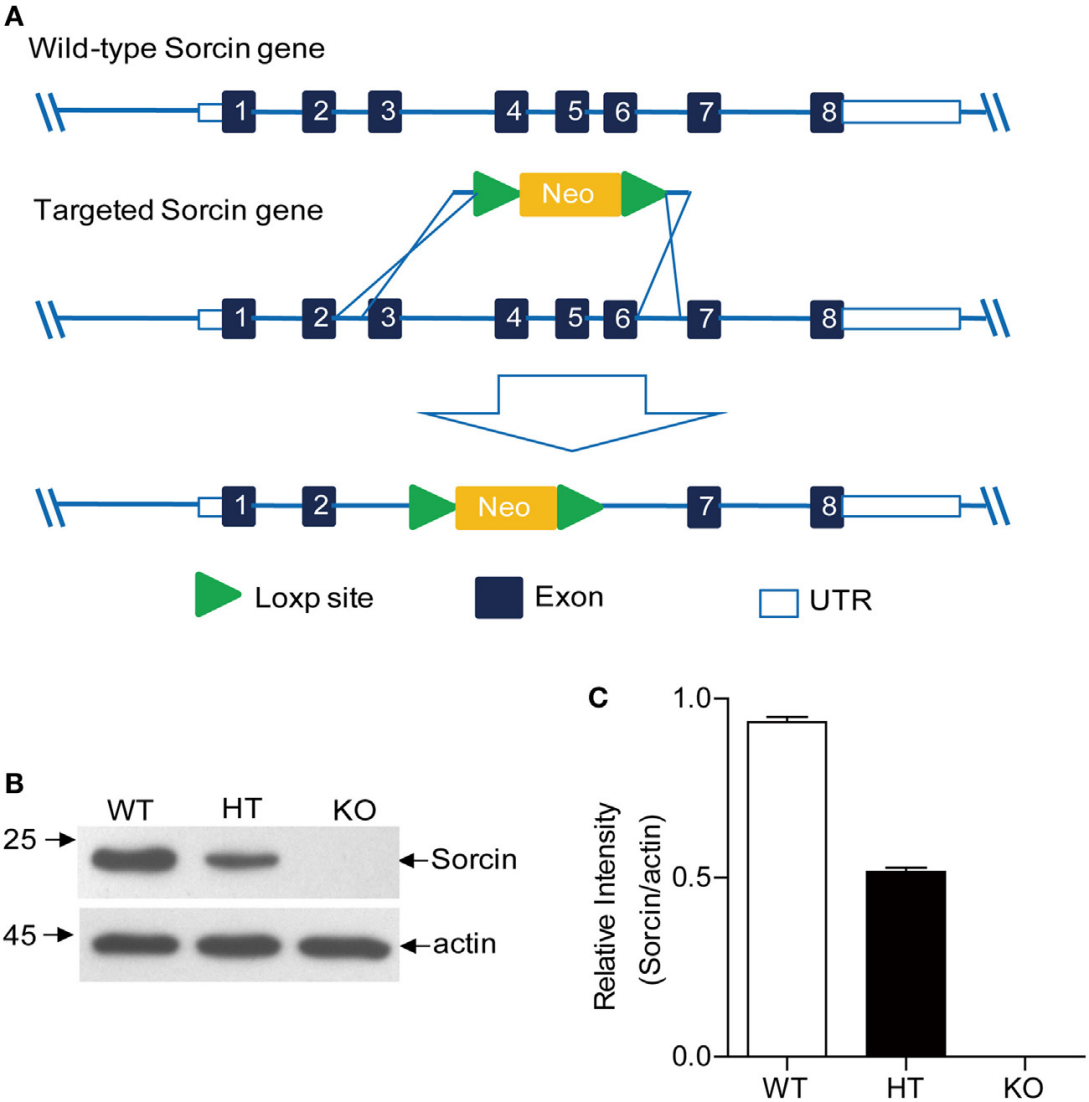
Generation of sorcin^−/−^ mice. **(A)** Homologous recombination strategy. The neomycin cassette served as the positive selection marker during the ES targeting step. Exons 3–6 were deleted by replacing exon 3–6 (~4.5 kb) with a loxp-pgkneo-loxp cassette. Two ES cell clones were identified by Southern Blot, which had one copy of the *sorcin* gene replaced by the neomycin resistance gene cassette. The mutant ES cells were injected into 4-day-old C57BL/6J mouse blastocysts. The resultant chimeric male offsprings were crossed with 129S6/SvEvTac females for germline transmission. **(B)** Examination of sorcin expression in splenocytes from sorcin^+/+^ (WT), sorcin^+/−^ (HT), or sorcin^−/−^ (KO) mice by Western Blot. Splenocytes were isolated from the freshly harvested spleens of sorcin^+/+^, sorcin^+/−^, and sorcin^−/−^ mice and lysed with lysis buffer, followed by examination with Western Blot using anti-sorcin antibodies. Endogenous β-actin expressions were used as internal controls. **(C)** The density of bands in **(B)** was quantitated by densitometry. The relative levels of sorcin were calculated as follows: the band density of sorcin/the band density of actin in the same sample. Results are representative of three independent experiments. Error bars are presented as mean ± SD, *n* = 3 samples.

Our previous report shows that sorcin inhibits type I interferon response in cells, suggesting that sorcin serves as an inhibitor for cell response (3). To investigate the roles of sorcin *in vivo*, we generated sorcin-deficient mice by deleting exons 3–6 of sorcin through homologous recombination (Figure 1A). The founders were backcrossed with C57BL/6 or BALB/c for over 10 generations to generate sorcin^−/−^ C57BL/6 or BALB/c mice. The sorcin protein is completely absent in mice homologous for the gene mutation (Figures 1B,C). Mice homozygous for the sorcin gene mutation developed normally and were born with the expected Mendelian ratio.

**Figure 2:**
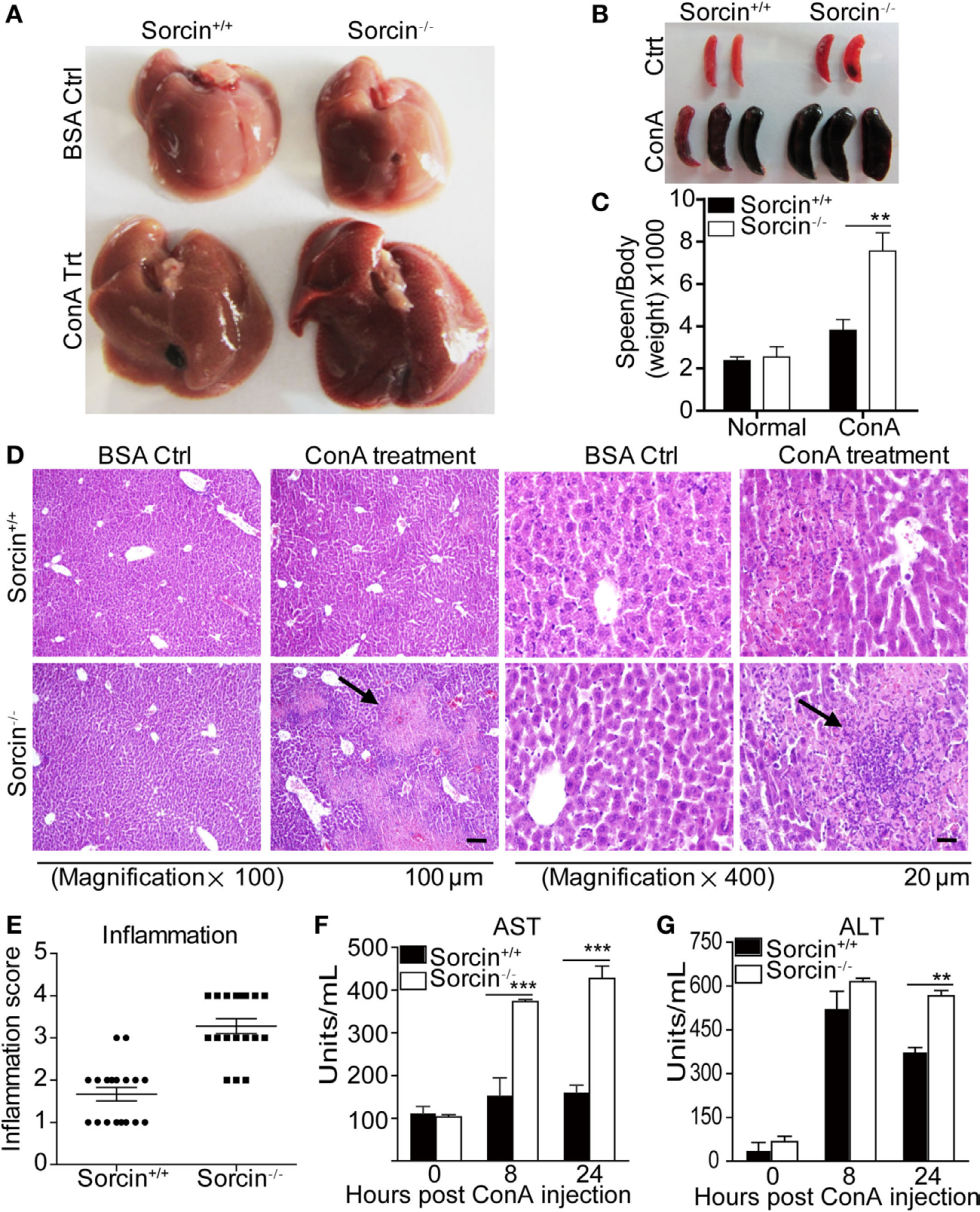
Enhanced ConA-induced hepatitis in sorcin^−/−^ mice. **(A)** Enhanced inflammation in the liver of sorcin^−/−^ mice post ConA treatment. Sorcin^−/−^ mice (*n* = 9) and the littermate WT controls (sorcin^+/+^) (*n* = 9) were injected with a single dose of ConA (20 mg/kg of body weight) to induce hepatitis as described in Section “Materials and Methods.” Mice were sacrificed 24 h post-ConA injection. The livers were harvested and pictures of the organs were taken. **(B,C)** Enhanced spleen mass in sorcin^−/−^ mice. Mice were treated with ConA as in **(A)**, and the spleens were harvested and weighed **(B)**, and the spleen weight vs. body weight of the same mouse was calculated **(C)**. **(D)** H&E straining of liver section from sorcin^+/+^ and sorcin^−/−^ mice, respectively, 24 h after ConA injection. Mice were treated with ConA as in **(A)**. The arrowhead indicated massive hepatic cell death and inflammatory infiltrates in sorcin^−/−^ liver sections. Original magnification, ×100 or ×400. The scale bars represent 20 or 100 µm, respectively. **(E)** Quantitative analysis of inflammatory lesions in the liver. Mice were treated with ConA as in **(A)**, and the degree of inflammation in the liver was graded as described in Section “Materials and Methods.” Lesions in cross sections per organ were examined. Each dot in the graph represents the average of lesions from the six continuous cross sections of each liver. Results shown are from a total of 18 mice. **(F,G)** Serum transaminase in ConA-induced hepatitis. Sorcin^+/+^ and sorcin^−/−^ mice were treated with ConA as in **(A)**. Serum transaminase levels were determined 8 and 24 h after the ConA injection. The ALT and aspartate aminotransferase (AST) levels are presented in Carmen’s units. Results are representative of three independent experiments with similar results. Error bars are presented as mean ± SD. The differences between the two groups are statistically significant as determined by two-way ANOVA (** stands for *p* < 0.01, and *** for *p* < 0.001).

To examine the potential roles of sorcin in inflammatory response *in vivo*, we induced hepatitis in mice with ConA. Sorcin^+/+^ and sorcin^−/−^ mice were injected intravenously with ConA, and hepatitis in mice was measured by anatomical and histological examinations of the liver and blood transaminase assays. As shown in Figures 2A,D, sorcin^−/−^ mice developed severe hepatitis post injection with ConA, which was characterized by widespread lesions, inflammatory infiltrates, and hepatocyte death. In contrast, sorcin^+/+^ displayed mitigated hepatitis with the same treatment. And in Figures 2B,C, the spleen of sorcin^−/−^ mice developed more serious hemorrhage, and more gain in weight. The histological analysis of lesions in the liver also revealed the increase of inflammatory infiltrates in sorcin^−/−^ mice (Figure 2E). Similarly, the results from serum transaminase assay showed markedly higher levels of ALT and AST in sorcin^−/−^ mice than in sorcin^+/+^ mice (Figures 2F,G). These results indicate that sorcin plays an inhibitory role in host inflammatory response *in vivo*.

### Enhanced Expressions of Proinflammatory Cytokines in Sorcin^−/−^ Splenocytes

**Figure 3:**
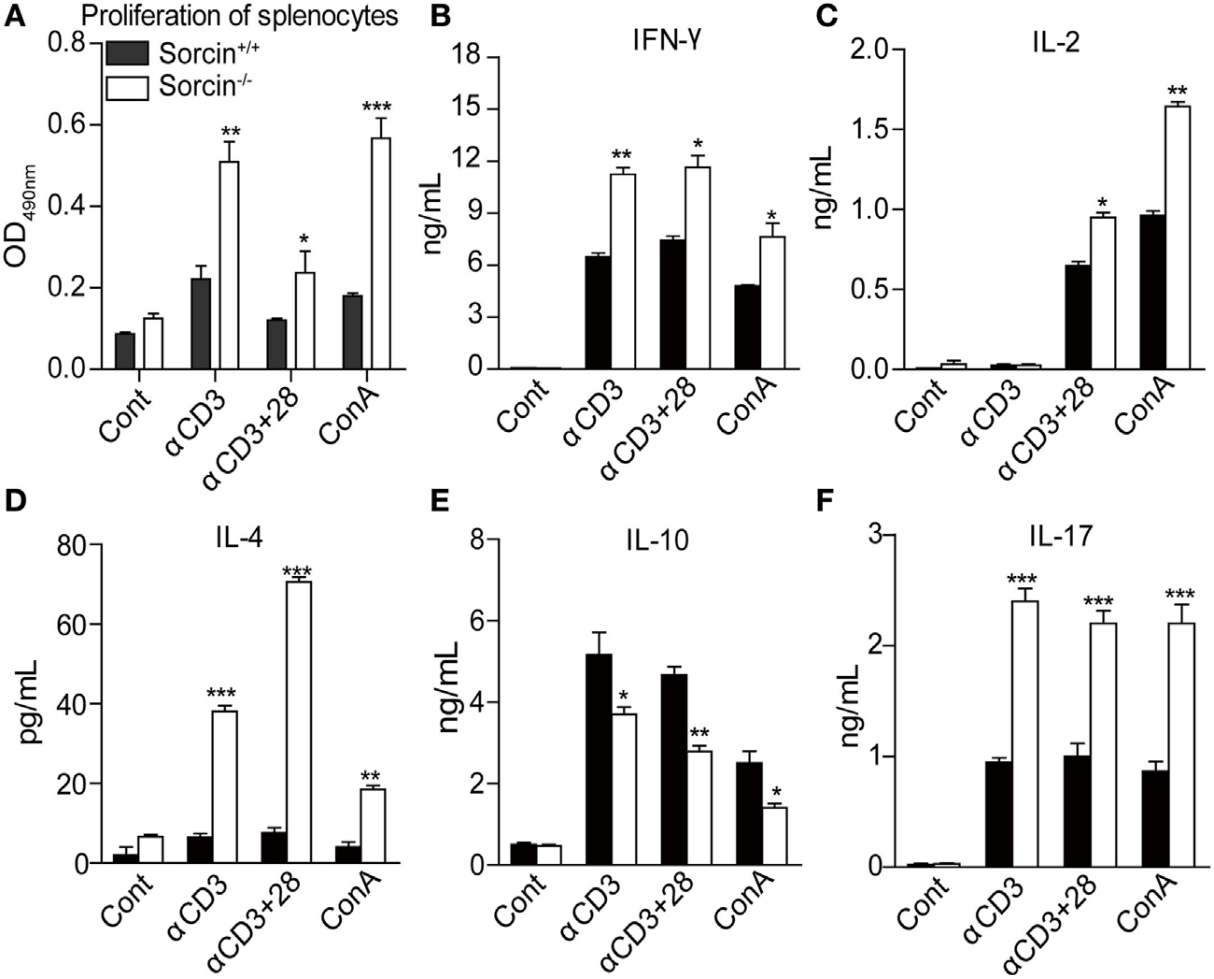
Enhanced T cell response in sorcin^−/−^ mice. **(A)** Splenocytes from sorcin^+/+^ (*n* = 4) and sorcin^−/−^ mice (*n* = 4) were stimulated with plate-bound anti-CD3 with or without soluble anti-CD28 McAbs or with Con A. Ninety-six hours post stimulation, the proliferation of splenic cells was measured by MTT assay. **(B–F)** Splenocytes from sorcin^+/+^ and sorcin^−/−^ mice were stimulated with plate-bound anti-CD3 with or without soluble anti-CD28 McAbs or with Con A. Forty-eight hours post stimulation, the culture supernatants were collected and cytokine concentrations were determined by CBA. **(A–F)** Results are representative of three independent experiments with similar results. Error bars are presented as mean ± SD. The differences between the two groups are statistically significant as determined by two-way ANOVA (* stands for *p* < 0.05, ** for *p* < 0.01, and *** for *p* < 0.001).

Since cytokines play key roles in host inflammatory response, we set out to examine the proliferation and cytokine expressions of splenocytes from sorcin^−/−^ mice. We cultured the sorcin^−/−^ or sorcin^+/+^ splenocytes with plate-bound anti-CD3 mAb in the presence or absence of anti-CD28 mAb or with ConA only, and measured the proliferation of cultured cells and cytokines in the supernatants of cell cultures. As a result, the proliferation of sorcin^−/−^ splenocytes was remarkably enhanced compared to that of sorcin^+/+^ controls (Figure 3A), and sorcin^−/−^ splenocytes produced more IL-2, IFN-γ, IL-4, and IL-17 but less IL-10 than the sorcin^+/+^ controls (Figures 3B–F). These data suggest that sorcin is involved in regulation of cytokine expressions in cells.

### Interaction of Sorcin with STAT3

**Figure 4:**
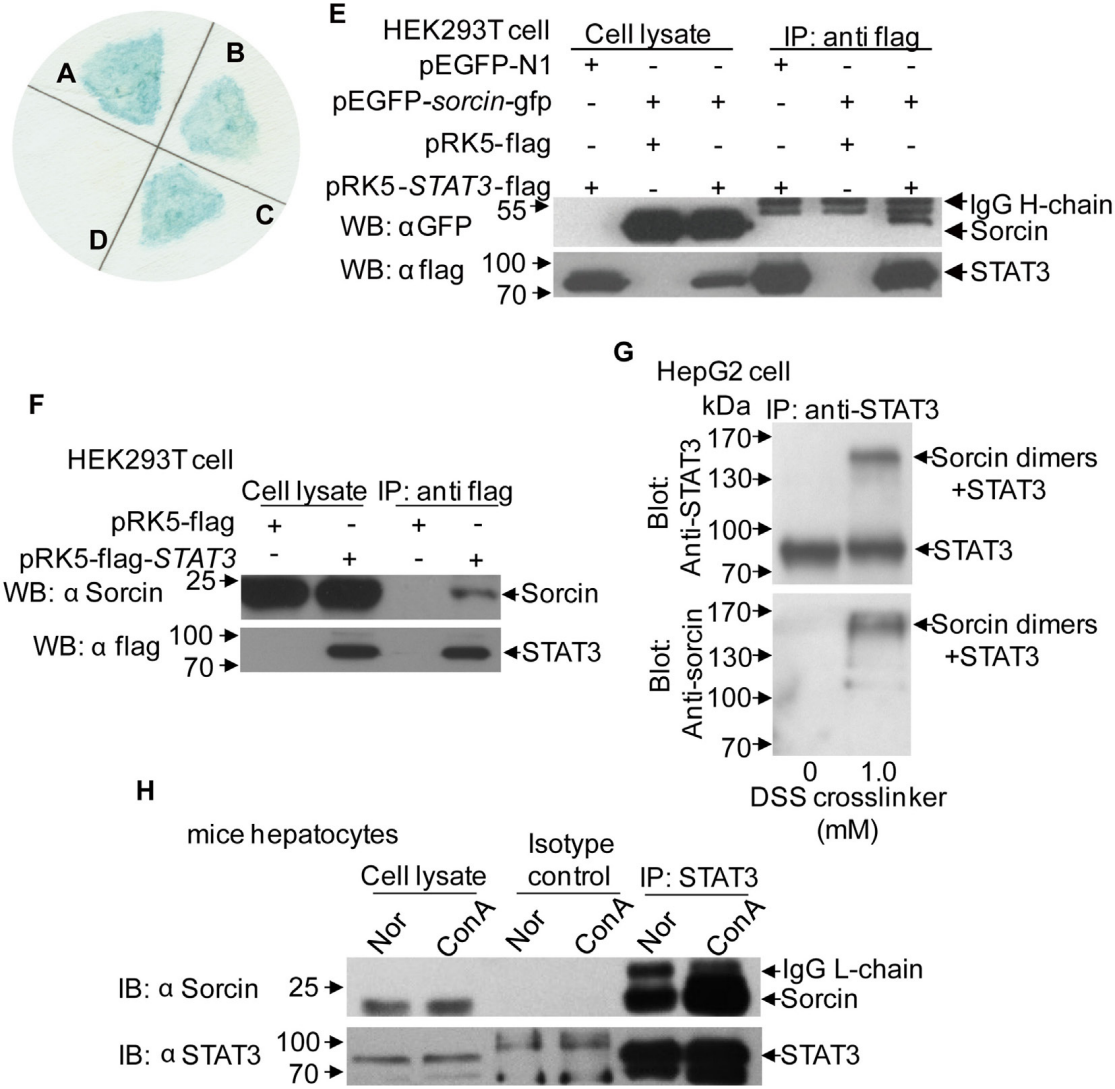
The interaction of sorcin with signal transducer and activator of transcription 3 (STAT3). **(A–D)** Yeast two-hybrid screening of sorcin-associated proteins. **(A)** pGBKT7-*p53* + pGADT7-*T* antigen as positive control. **(B)** pGBKT7-*sorcin* + pGADT7-*lectin*. **(C)** pGBKT7-*sorcin* + pGADT7-*STAT3*. **(D)** pGBKT7-*Lamin C* + pGADT7-*T* antigen as negative control. **(E)** The interaction of sorcin with exogenous STAT3. HEK293T cells (8 × 10^5^/well) were transfected with 2.5 µg of pEGFP-*sorcin* and/or pRK5-flag-*STAT3* or empty vector as controls, and co-immunoprecipitation assay was performed with anti-flag monoclonal antibody. Sorcin and STAT3 in the immune complex were examined with Western Blot using anti-GFP and anti-flag monoclonal antibodies, respectively. **(F)** The interaction of STAT3 with endogenous sorcin in HEK293T cells. HEK293T cells were transfected with 5 µg of pRK5-flag-*STAT3* or empty vector, and co-immunoprecipitation assay was performed with anti-flag monoclonal antibody. Sorcin in the immune complex was examined with Western Blot using anti-sorcin monoclonal antibody. **(G)** Chemical crosslinking of endogenous STAT3 and endogenous sorcin. HepG2 cells were cultured, washed with PBS and treated with disuccinimidyl suberate (DSS) as described in Section “Materials and Methods.” Cell lysates were co-immunoprecipitated with anti-STAT3 antibody. After SDS-PAGE and blotted onto nitrocellulose, the blots were probed with anti-STAT3 (top) or anti-sorcin (bottom) antibodies. **(H)** Endogenous sorcin interacts with endogenous STAT3 in mice hepatocytes. Mice were treated with ConA as in Figure 2A. The hepatocytes were separated from mice with or without ConA stimulation. The hepatocyte extracts were immunoprecipitated with STAT3 antibody and control IgG. After SDS-PAGE and blotted onto PVDF, the blots were probed with anti-STAT3 or anti-sorcin antibodies. **(A–H)** Results are representative of three independent experiments with similar results.

To investigate the mechanism of how sorcin regulates the expressions of proinflammatory cytokines in cells, we performed yeast two-hybrid assay to screen human leukocyte cDNA library using full-length human sorcin as bait. Our results showed that one sorcin-interacting protein was STAT3 (Figures 4A–D). To confirm the interaction of sorcin with STAT3, we performed an immunoprecipitation assay. As shown in Figure 4E, sorcin and STAT3 interacting complex was precipitated from pRK5-flag-*STAT3* and pEGFP-*sorcin* plasmid transfected cells but not from those transfected with either pRK5-flag-*STAT3* or pEGFP-*sorcin* plasmid plus empty vectors. Furthermore, when cells were transfected with pRK5-flag-*STAT3* or pRK5-flag empty vector, sorcin could only be pulled down from the lysates of cells transfected with pRK5-flag-*STAT3* but not from the empty vector transfected controls (Figure 4F), suggesting that sorcin interacts with STAT3 in host cells. Importantly, chemical crosslinking of endogenous STAT3 and sorcin by DSS allowed immunoprecipitation of STAT3–sorcin complex by anti-STAT3 antibodies, and detection of sorcin by Western Blot, which directly confirmed the interaction of sorcin with STAT3 in cells (Figure 4G). In addition, we examined the interaction of endogenous sorcin with endogenous STAT3 in mice hepatocytes separated from mice with or without ConA stimulation. Likewise, endogenous sorcin was pulled down by anti-STAT3 antibodies, but not from IgG isotype controls (Figure 4H). These results clearly show that sorcin interacts with STAT3 in host cells.

### Domains That Span Residuals 1–100 of Sorcin and Residuals 580–770 of STAT3 Are Involved in Sorcin–STAT3 Interaction

**Figure 5:**
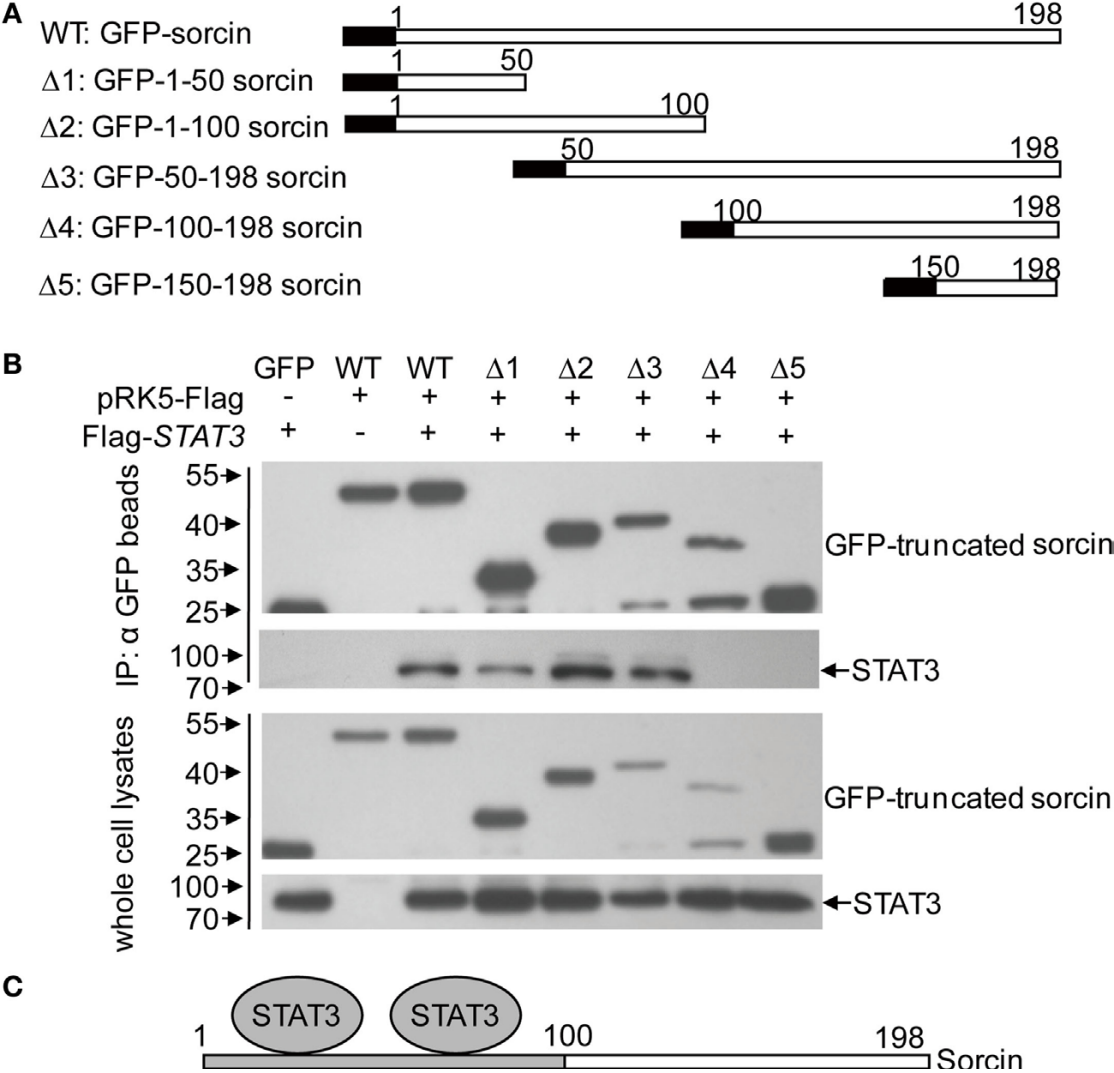
Determination of the binding domains of sorcin for signal transducer and activator of transcription 3 (STAT3). **(A)** Schematics represent the genes encoding the full length and truncated sorcin molecules. **(B)** STAT3 interacts with different truncated sorcin proteins. HEK293T cells were transfected with the full length or truncated sorcin plasmids or empty vectors and together with the full-length flag-STAT3. Twenty-four hours after transfection, cell lysates were immunoprecipitated by anti-GFP monoclonal antibodies. The pellets were subjected to Western Blot analysis using anti-flag monoclonal antibody. Results are representative of three independent experiments with similar results. **(C)** A schematic represents the sorcin-binding domain (aa 1–100 of sorcin) for STAT3.

**Figure 6:**
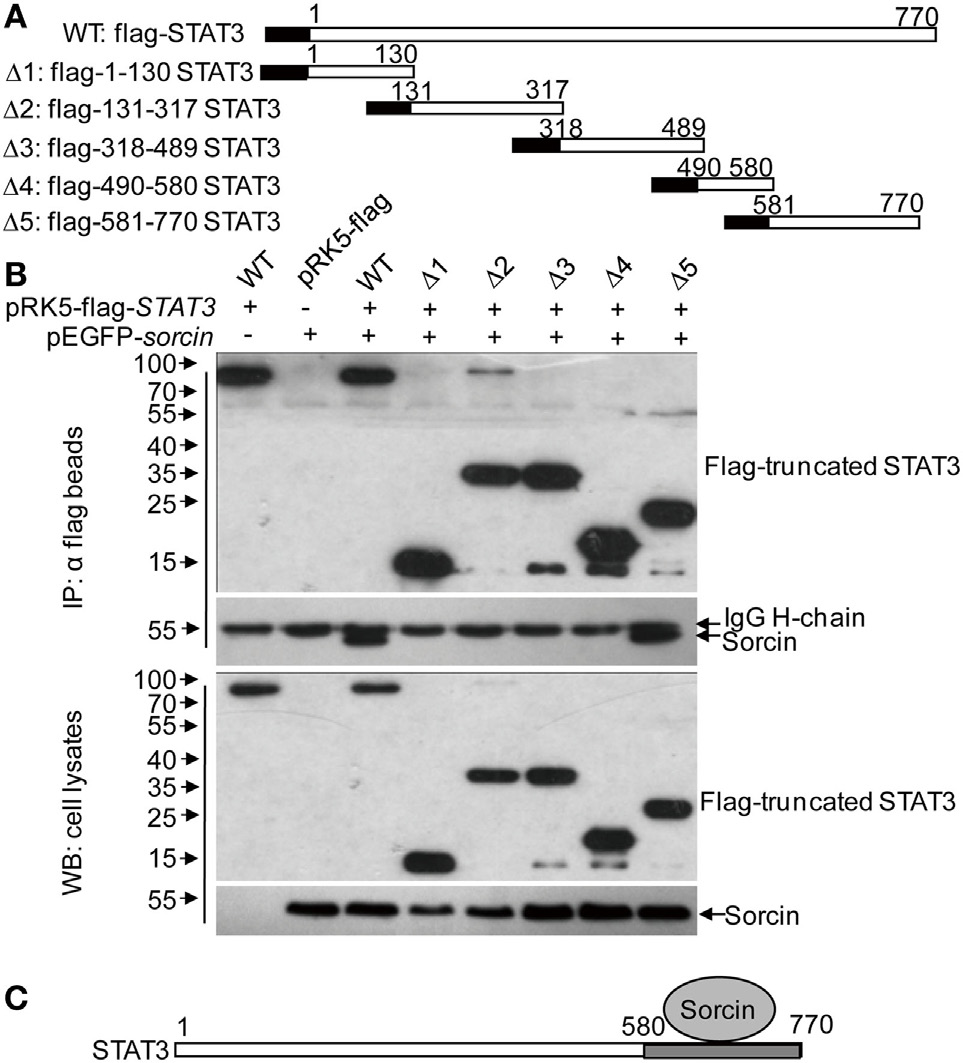
Determination of the binding domain of signal transducer and activator of transcription 3 (STAT3) for sorcin. **(A)** Schematics represent the genes encoding the full length and truncated STAT3 molecules. **(B)** Sorcin interacts with different truncated STAT3 proteins. HEK293T cells were transfected with the full length or truncated STAT3 plasmids or empty vectors and together with pEGFP-*sorcin*. Twenty-four hours after tansfection, cell lysates were immunoprecipitated by anti-flag monoclonal antibodies. The pellets were subjected to Western Blot analysis using anti-GFP monoclonal antibody. Results are representative of three independent experiments with similar results. **(C)** A schematic represents the binding domain of STAT3 (aa 581–770 of STAT3) for sorcin.

To determine the region of sorcin responsible for interaction with STAT3, we made a series of constructs with sorcin deletion mutants fused to the GFP tag (Figure 5A). These sorcin derivatives were individually expressed in HEK293T cells, and their interactions with STAT3 were determined by immunoprecipitation. Our results showed that except for mutant Δ4, Δ5, the sorcin mutants including Δ1, Δ2, andΔ3 retained the ability to interact with STAT3 (Figure 5B), indicating that the region of 1–100 sorcin is important for its interaction with STAT3 (Figure 5C). Using the similar strategy, we constructed a series of STAT3 deletion mutants fused to the Flag tag (Figure 6A). These STAT3 derivatives were individually expressed in HEK293T cells and their interactions with sorcin were determined by immunoprecipitation. Our results indicated that only the mutant with residuals 581–770 interacted with sorcin (Figure 6B), indicating that this region of STAT3 interacts with sorcin (Figure 6C). These data clearly show that domains that span residuals 1–100 of sorcin and residuals 580–770 of STAT3 are involved in sorcin–STAT3 interaction. The determination of the binding domains in sorcin and STAT3 further confirmed their interaction.

### STAT3 Acts As an Immediate Downstream Molecule of Sorcin in the Negative Regulation of TNF-α-Induced NF-κB Signaling

**Figure 7:**
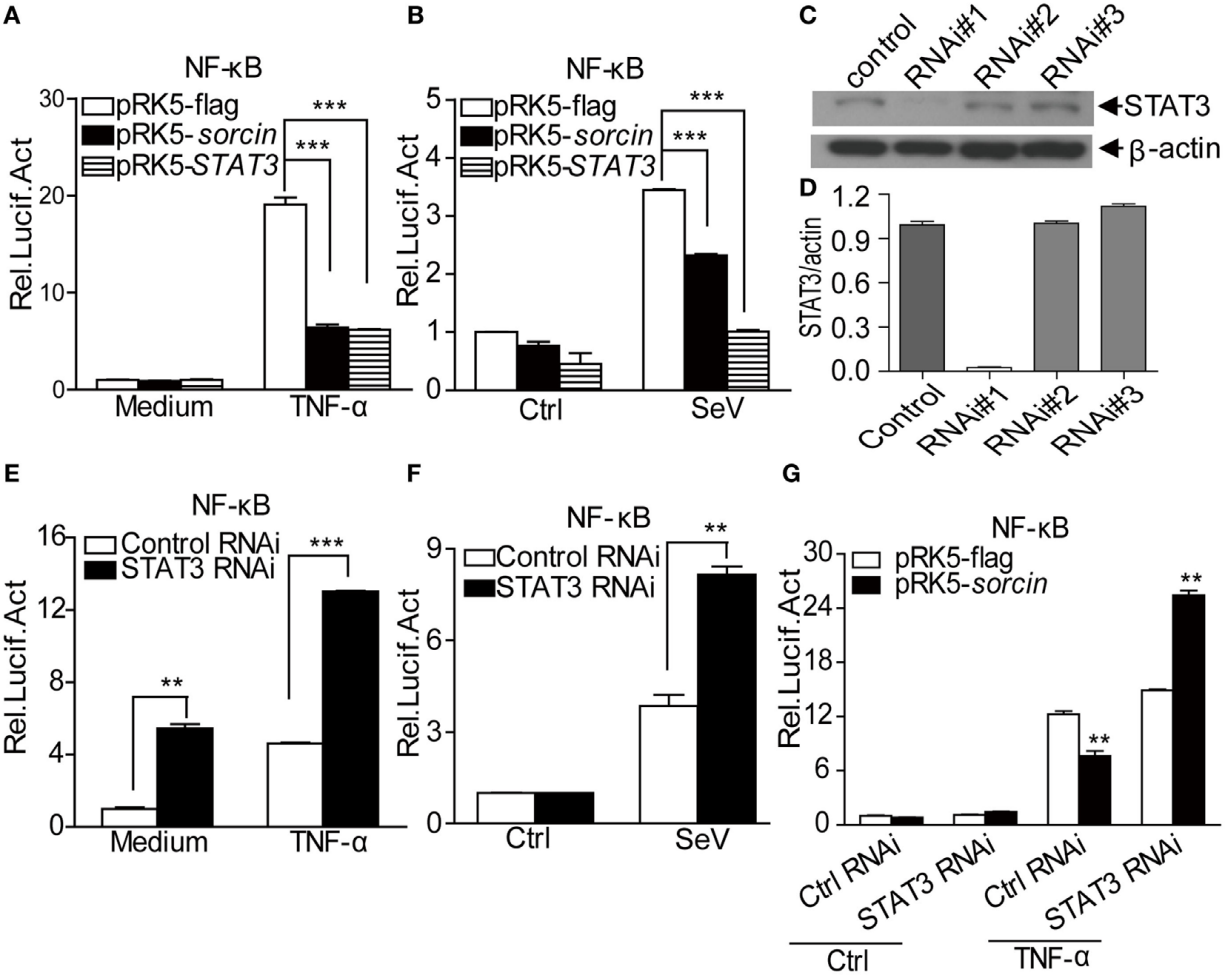
Roles of sorcin and signal transducer and activator of transcription 3 (STAT3) in TNF-α- or SeV-induced activation of NF-κB promoters. **(A,B)** Overexpression of sorcin or STAT3 inhibits TNF-α- or SeV-induced activation of NF-κB promoters. HEK293T cells were transfected with pRK5-*sorcin* or pRK5-*STAT3* or empty vector together with indicated reporter plasmids. Eighteen hours after transfection, cells were treated with TNF-α or SeV. After treatment, the activities of NF-κB promoter were determined by luciferase assays. **(C,D)** Effects of STAT3 RNAi on the expression of endogenous STAT3. HEK293T cells were transfected with siRNA (#1–3) or controls as described in Section “Materials and Methods.” Twenty-four hours after the second transfection, cell lysates were prepared and examined by Western Blot with anti-STAT3 antibody **(C)**. Endogenous β-actin expressions were used as internal controls. The density of bands in **(C)** was quantitated by densitometry **(D)**. The relative levels of STAT3 in STAT3 RNAi cells were calculated as follows: the ratio (the band density of STAT3/that of actin in the same sample) of STAT3 RNAi/the ratio (the band density of STAT3/that of actin) of RNAi control. **(E,F)** TNF-α- or SeV-induced activation of NF-κB promoters were enhanced in STAT3 knockdown cells. HEK293T cells were transfected with the STAT3 RNAi construct or RNAi control together with indicated reporter plasmids. Eighteen hours after transfection, cells were treated as in **(A,B)**. **(G)** Knockdown of STAT3 abolished sorcin suppressed activation of NF-κB promoters upon TNF-α treatment. HEK293T cells were transfected with STAT3 RNAi construct or RNAi control together with pRK5-flag-*sorcin* or empty vector and indicated reporter plasmids. Eighteen hours after transfection, cells were treated as in **(A,B)**. **(A,B,D,E–G)** Results are representative of three independent experiments with similar results. Error bars are presented as mean ± SD, *n* = 3 cultures. The significance of difference between the groups was performed by two-way ANOVA (** stands for *p* < 0.01, and *** for *p* < 0.001).

Since STAT3 interacted with sorcin and our previous results showed that sorcin negatively regulated TNF-α-induced NF-κB signaling (3), this information prompted us to investigate the role of STAT3 in TNF-α-induced NF-κB signaling. We transfected HEK293T cells with pRK5-flag-*STAT3* or pRK5-flag-*sorcin* plasmids and examined the effects of STAT3 on TNF-α- or SeV-induced activation of NF-κB promoters. As shown in Figure 7A, like sorcin, overexpression of STAT3 markedly inhibited TNF-α-induced activation of NF-κB promoter (*p* < 0.001). Similarly, overexpression of STAT3 also inhibited SeV-induced activation of NF-κB promoter (*p* < 0.001) (Figure 7B). To further examine the role of STAT3 in sorcin-mediated negative regulation of TNF-α- or SeV-induced activation of NF-κB promoters, we made three STAT3 RNAi constructs and found that the RNAi#1 construct could effectively inhibit the expression of endogenous STAT3 (Figures 7C,D). Therefore, the RNAi#1 construct was used to knockdown STAT3 in the following assays. In contrast to the inhibitory effect of epitopically expressed STAT3 on NF-κB signaling, knockdown of STAT3 markedly enhanced TNF-α- or SeV-induced activation of NF-κB promoters (Figures 7E,F). These data indicate that STAT3 is involved in negative regulation of TNF-α- or SeV-induced activation of NF-κB promoter. Importantly, knockdown of STAT3 completely reversed the inhibitory effect of sorcin on TNF-α-induced activation of NF-κB promoter (*p* < 0.01) (Figure 7G). Since sorcin interacts with STAT3, these results indicate that STAT3 is a signal transduction molecule immediate downstream of sorcin in the TNF-α-induced NF-κB signaling. Thus, sorcin suppressed TNF-α-induced activation of NF-κB promoters *via* STAT3.

### Sorcin Enhances Phosphorylation of STAT3 on Tyr705 in Hepatoma Cells with IL-6 Treatment

**Figure 8:**
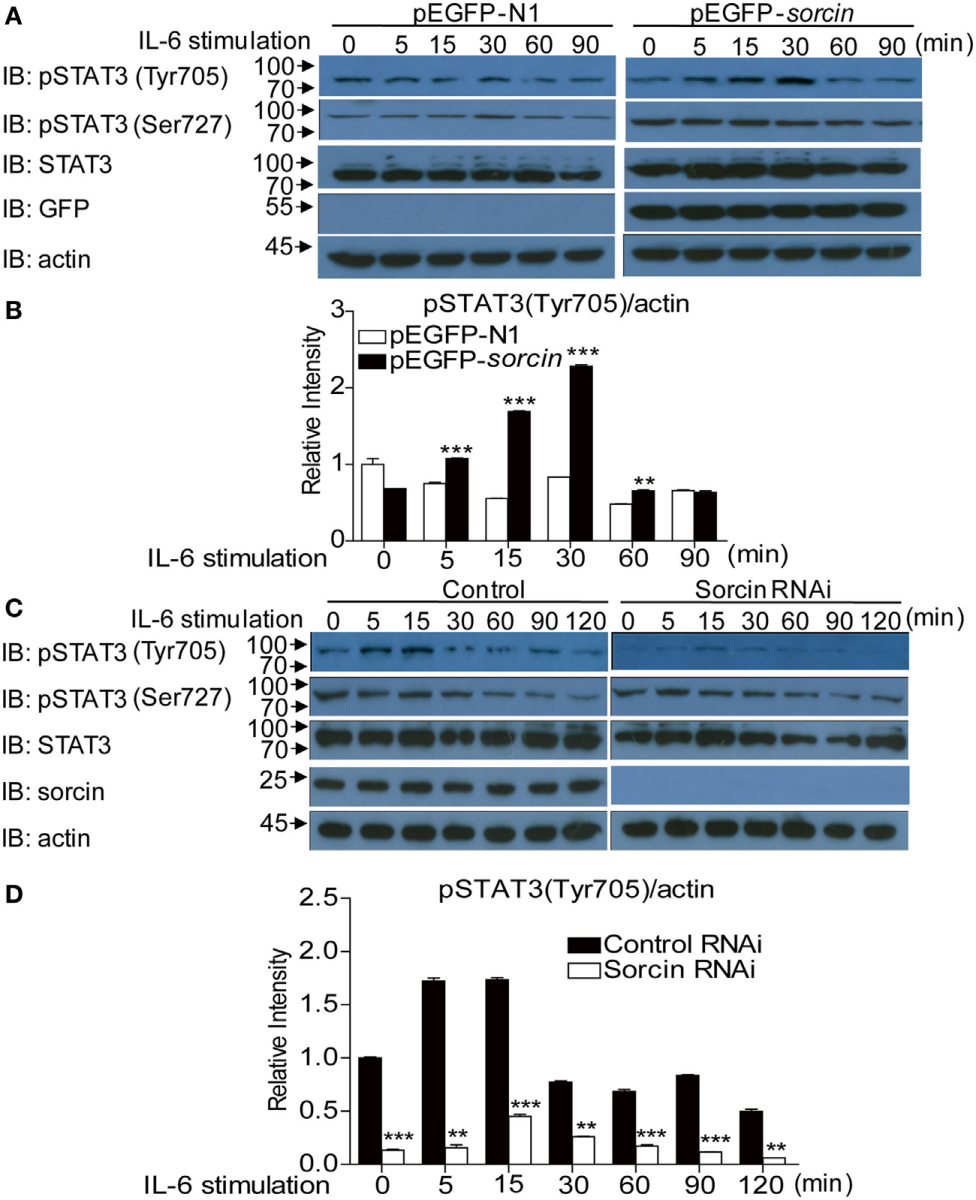
Overexpression or knockdown of sorcin influences phosphorylation of signal transducer and activator of transcription 3 (STAT3) on Tyr705 in hepatoma cells. **(A)** Overexpression of sorcin enhances phosphorylation of STAT3 on Tyr705 but not Ser727 in hepatoma cells. Hep2G cells were transfected with pEGFP-N1 or pEGFP-*sorcin*, and the cells were stimulated with IL-6 (20 ng/mL) for the indicated time when cells were lysed and subjected to Western Blot analysis using anti-pSTAT3 (Tyr705), anti-pSTAT3 (Ser727), anti-STAT3, anti-GFP, and anti-actin antibodies. **(B)** Densitometric quantification of the results in **(A)** was shown. The relative intensity of pSTAT3 (Tyr705) was normalized to the endogenous actin of the same sample. **(C)** Knockdown of sorcin reduced phosphorylation of STAT3 on Tyr705 but not Ser727 in hepatoma cells. Hep2G cells were transfected with s*orcin* RNAi constructs or RNAi controls. Twenty-four hours after the second transfection, cells were stimulated with IL-6 (20 ng/mL) for the indicated time, and cells were lysed and subjected to Western Blot analysis using with anti-pSTAT3(Tyr705), anti-pSTAT3(Ser727), anti-STAT3, anti-sorcin, or anti-actin antibodies. **(D)** Densitometric quantification of the results in **(C)** was shown. Relative intensity of pSTAT3 (Tyr705) was normalized to the endogenous actin of the same sample. **(B,D)** Results are representative of three independent experiments with similar results. Error bars are presented as mean ± SD, *n* = 3 cultures. The significance of difference between the groups was performed by two-way ANOVA (** stands for *p* < 0.01, and *** for *p* < 0.001).

**Figure 9:**
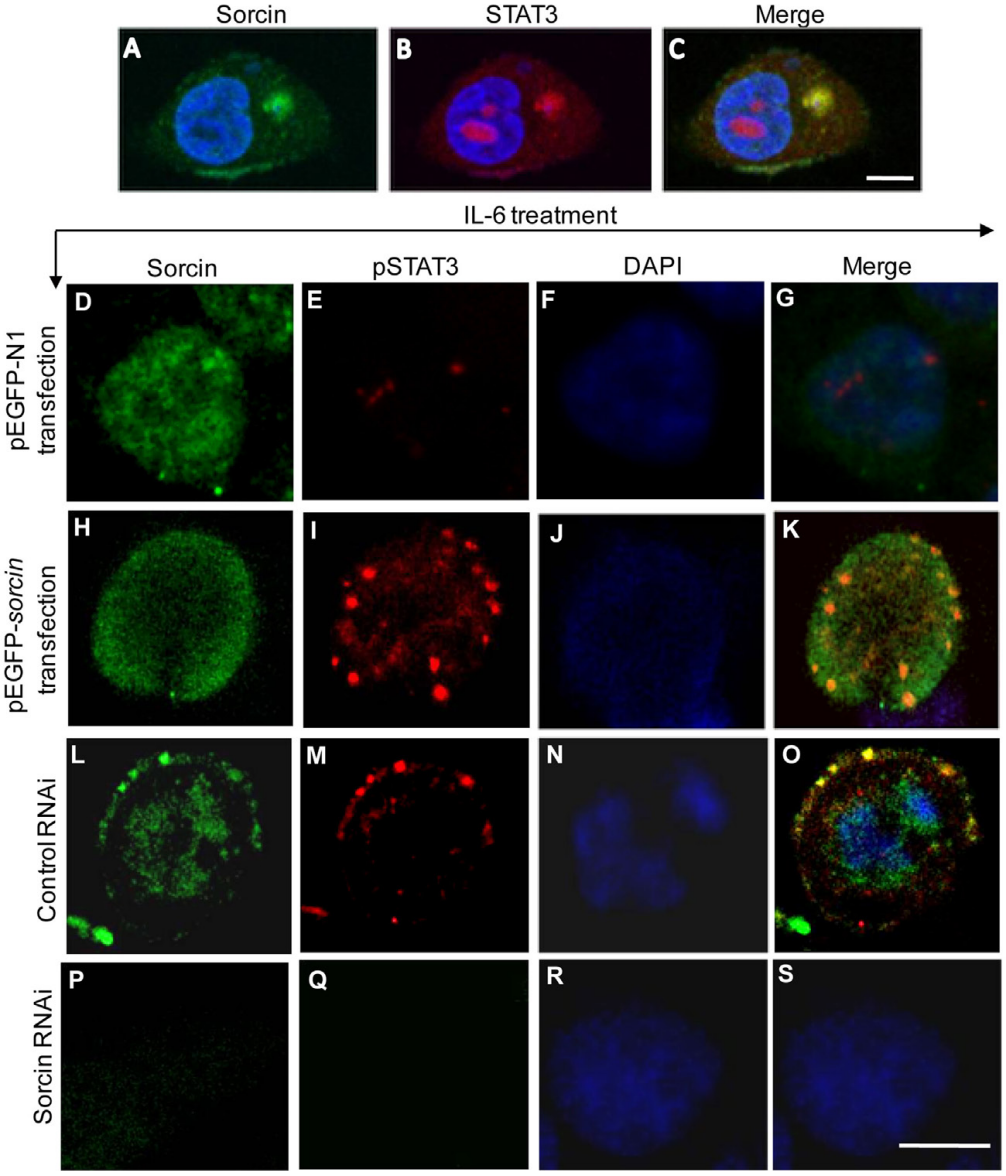
Colocalization of sorcin with signal transducer and activator of transcription 3 (STAT3). **(A–C)** Colocalization of sorcin with STAT3 in normal HepG2 cells. HepG2 cells were fixed and probed with mouse anti-sorcin antibody and rabbit anti-STAT3 antibody, followed by the FITC-conjugated goat anti-mouse antibody (green) and TRITC-conjugated goat anti-rabbit antibody (red). Nuclei were counterstained with DAPI (blue). The cell samples were observed with a laser confocal scanning microscope. All scale bars represent 10 µm. **(D–K)** Overexpression of sorcin enhanced IL-6-induced phosphorylation of STAT3. HepG2 cells were transfected with the pEGFP-N1 **(D–G)** or pEGFP-*sorcin* **(H–K)**, and the cells were stimulated with IL-6 (20 ng/mL) for 30 min. Then the cells were fixed with 1% paraformaldehyde. After washes, the fixed cells were permeabilized with 0.1% triton X-100, and probed with anti-sorcin and anti-pSTAT3 (Tyr705) antibodies, and followed by the FITC- and TRITC-conjugated secondary antibodies. Nuclei were counterstained with DAPI (Blue). **(L–S)** Knockdown of sorcin suppressed IL-6-induced phosphorylation of STAT3. HepG2 cells were transfected with the control RNAi or sorcin RNAi construct, and the cells were stimulated with IL-6 (20 ng/mL) for 30 min. Then the cells were fixed with 1% paraformaldehyde. After washes, the fixed cells were permeabilized with 0.1% triton X-100, and probed with anti-sorcin and anti-pSTAT3 (Tyr705) antibodies, and followed by the FITC- and TRITC-conjugated secondary antibodies. Nuclei were counterstained with DAPI (Blue). All scale bars represent 10 µm. **(A–S)** Results are representative of three independent experiments with similar results, and for each experiment, 50 cells were observed.

In the early response to pathogenic infection, the monocytes of host immune system recognize pathogen-associated molecular patterns (PAMPs) *via* pattern recognition receptors (PRRs) and produce varied proinflammatory cytokines including IL-6. Engagement of IL-6 with its receptors initiates immune signaling transduction in cells that requires phosphorylation of STAT3 (17). To determine whether sorcin affects phosphorylation of STAT3, we transfected cells with pEGFP-*sorcin* and examined the phosphorylation of STAT3 in these cells after treatment with IL-6 at different time points. As a result, expression of sorcin-GFP fusion significantly enhanced phosphorylation of STAT3 on Tyr705 amino acid residual but not on Ser727 (Figures 8A,B). On the contrary, knockdown of sorcin markedly reduced phosphorylation of STAT3 on Tyr705 (Figures 8C,D). Furthermore, we employed confocal microscopy to examine the effect of sorcin on phosphorylation of STAT3 in cells after IL-6 treatment. As shown in Figures 9A–C, both sorcin and STAT3 formed speckles in normal cells and they were well colocalized, further suggesting that sorcin interacted with STAT3. When cells were treated with IL-6, the phosphorylation of STAT3 on Tyr705 in pEGFP-*sorcin* transfected cells dramatically increased compared to empty vector transfected controls (Figures 9D–K). In contrast, knockdown of sorcin completely abolished phosphorylation of STAT3 (Figures 9L–S). These results indicate that sorcin exerts inhibitory effects on NF-κB signaling by interacting with STAT3 and enhancing its phosphorylation.

## Discussion

Sorcin was originally identified in drug-resistant cells (13, 14). It belongs to the penta-EF hand (PEF) family of calcium-binding proteins (18). It was found that sorcin interacts with multiple proteins such as the cardiac ryanodine receptor (19), the pore-forming subunit of voltage-dependent L-type Ca^2+^ channels (20), Presenilin 2 (21), grancalcin (18), the calcium/calmodulin-dependent protein kinase II C (22), sodium–calcium exchanger (NCX1) (23), tumor necrosis factor receptor-associated protein 1 (Trap1) (15), carbohydrate-responsive element-binding protein (24), Polo-like kinase 1 (25), and PDCD6 (26), suggesting that sorcin might be a multifunctional cellular protein. Sorcin was involved in regulation of myocardial cell excitation–contraction coupling (19, 20), multi-drug resistance in tumor cells (13, 27, 28), and cytoprotection (15). Most recently, sorcin has been found to link pancreatic β-cell lipotoxicity to ER Ca^2+^ stores, affecting glucose tolerance in type 2 diabetes (29). Furthermore, our previous work and the work by another group show that sorcin interacts with some viral proteins and is associated with virus replication and cell response (3, 30). These findings indicate that sorcin is involved in multiple physiological processes.

In our previous study, our data show that FMDV VP1 suppresses type I interferon expression *via* interaction with sorcin that negatively regulates cell response to virus infection (3). Recently, it was found that hepatitis C virus (HCV) NSP5A interacted with sorcin that was required for HCV propagation (30). These findings suggest a novel role for sorcin in the regulation of immune responses to host cells.

In the present study, we generated sorcin^−/−^ mice by homologous recombination, and the founders were backcrossed with C57BL/6 or BALB/c for over 10 generations to generate sorcin^−/−^ C57BL/6 or BALB/c mice. These sorcin^−/−^ mice did not show any abnormality in fertility, growth, and survival compared to their littermate controls, suggesting that sorcin is not a critical factor for the viability of animals. This information agrees with our previous findings that sorcin acts as a negative regulator for immune responses. Our data from the present study show that sorcin^−/−^ mice has enhanced inflammatory response to ConA-induced hepatitis and cytokine expressions, providing direct evidence to show that sorcin plays an important role as a negative regulator in immune response.

In this study, STAT3 was identified as a sorcin-interacting protein, which supported the previous finding by Wu’s group that STAT3 interacted with sorcin (31). We found that sorcin suppressed TNF-α- or SeV-induced NF-κB signaling *via* interaction with STAT3. Knockdown of the STAT3 abolished the inhibitory effects of sorcin on TNF-α-induced NF-κB signaling, suggesting that STAT3 functions immediately downstream of sorcin in TNF-α-induced NF-κB signaling pathway. Importantly, our data from Western Blot analysis show that sorcin regulates phosphorylation of STAT3 on Tyr705 in cells, and these results were further confirmed by the confocal microscopy assays. As phosphorylation of STAT3 is required for this molecule to play a role in cell response and STAT3 also opposes NF-κB-dependent immune responses (12), sorcin is very likely to suppress the NF-κB signaling *via* enhancing phosphorylation of STAT3.

STATs enhance transcription of specific genes in response to cytokines and growth factors (32). Cross talk between transcription factors has become a commonly recognized mode of gene regulation, for example, NF-κB cooperates with STAT6 (33), and STAT3 binds to c-Jun (34) and IL-6-induced tethering of STAT3 links to the LAP/C/EBPB promoter (35). It has been demonstrated that interleukin 1 activates STAT3/NF-κB, and the cross talk between NF-κB p65 and STAT3 contributes to cytokine-mediated activation of a DNA-binding site that has a higher relative affinity for p65 homodimers than does a classical κB motif (36). Our data show that STAT3, as an immediate downstream molecule of sorcin, is involved in the negative regulation of TNF-α-induced NF-κB signaling. As TNF-α is one of the earliest cytokines produced by mononuclear phagocytes in recognition of PAMPs *via* PRRs, TNF-α-induced NF-κB signaling further magnifies inflammatory responses. If the inflammatory response cannot be properly controlled, it may cause septic shock due to “cytokine storms,” leading the death of animals. Thus, sorcin may serve as a brake *via* interaction with STAT3 to slow down TNF-α-triggered inflammatory response to avoid excessive cytokine productions, which may cause self damage.

NF-κB serves as a molecular sensor that responds to environmental changes including those caused by viral infection. Some viruses, such as cytomegalovirus (37), human immunodeficiency virus (38), rhinovirus (39), Theiler’s murine encephalomyelitis virus (40), and measles virus (41), have been reported to activate NF-κB and induce an inflammatory and/or antiviral response. By contrast, engagement of viral proteins (FMDV VP1) with sorcin inhibits TNF-α-induced NF-κB signaling (3), thus antagonizing the host response. As NF-κB regulates many aspects of immune responses, inhibition of NF-κB signaling by sorcin *via* STAT3 may contribute to virus persistent infection such as FMDV infection.

In summary, our data show that sorcin^−/−^ animals display enhanced ConA-induced hepatitis and express more cytokines (IL-2, IL-4, IL-17, and IFN-γ) than the wild-type controls, indicating that sorcin acts as a negative regulator in immune response *in vivo*. Furthermore, our results indicate that sorcin interacts with STAT3 and that STAT3 acts as an immediate downstream molecule of sorcin in the negative regulation of NF-κB signaling. Thus, sorcin, in association with STAT3, negatively regulates immune responses.

## Ethics Statement

All procedures for animal experiments were approved by the Institutional Animal Care and Use Committee of China Agricultural University (Approval IDs: XXMB-2012-03-01-1, XXMBB-2012-03-15-1, and SKLAB-2016-01-06) and used in accordance with regulations and guidelines of this committee.

## Author Contributions

Conceived and designed the experiments: SZ; performed the experiments: XYL and YL; analyzed the data: XYL, YL, and SZ; contributed reagents/materials/analysis tools: SZ, YW, JL, XG, HC, and XQL; wrote the paper: SZ, XYL, and YL; other: teaching technique: SZ.

## Conflict of Interest Statement

The authors declare that the research was conducted in the absence of any commercial or financial relationships that could be construed as a potential conflict of interest.
