# Supplementary material for: Negative Regulation of Hepatic Inflammation by the Soluble Resistance-Related Calcium-Binding Protein via Signal Transducer and Activator of Transcription 3
Source: Front Immunol. 2017 Jun 29;8:709. doi: 10.3389/fimmu.2017.00709 (PMC5489593; doi:10.3389/fimmu.2017.00709)
Supplement: Supplementary file 1 [file Image_1.PDF]

Supplementary Figure 1

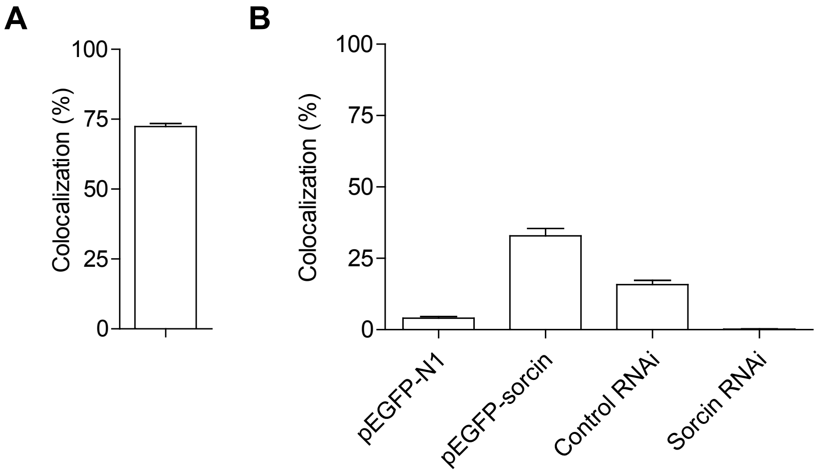

Supplementary Figure 1. Quantitative data of colocalization using Mander's coefficient. A. Plotted is the percentage of signal of sorcin that is also positive for STAT3 in normal HepG2 cells in Figure 9A-9C. B. Plotted is the percentage of signal of sorcin that is also positive for STAT3 in overexpression or knockdown of sorcin in HepG2 cells with IL-6 treatment in Figure 9D-9S. The software of Image J was used to analyze the results. Results are representative of three independent experiments with similar results, and for each experiment, 50 cells were observed.
